# Comparison of weight changes following unilateral and staged bilateral STN DBS for advanced PD

**DOI:** 10.1002/brb3.9

**Published:** 2011-09

**Authors:** Eric M Lee, Ashish Kurundkar, Gary R Cutter, He Huang, Barton L Guthrie, Ray L Watts, Harrison C Walker

**Affiliations:** 1 Research Associate, Department of Neurology, University of Alabama at BirminghamAlabama 35294-1150; 2 Department of Biostatistics, University of Alabama at BirminghamAlabama 35294-1150; 3 Division of Neurosurgery, Department of Surgery, University of Alabama at BirminghamAlabama 35294-1150; 4 Division of Movement Disorders, Department of Neurology, University of Alabama at BirminghamAlabama 35294-1150

**Keywords:** Bilateral, deep brain stimulation, Parkinson's disease, subthalamic, unilateral, weight

## Abstract

Unilateral and bilateral subthalamic nucleus deep brain stimulation (STN DBS) in Parkinson's disease (PD) result in weight gain in the initial postoperative months, but little is known about the changes in weight following unilateral and staged bilateral STN DBS over longer time intervals. A case–control comparison evaluated weight changes over 2 years in 43 consecutive unilateral STN DBS patients, among whom 25 elected to undergo staged bilateral STN DBS, and 21 age-matched and disease severity matched PD controls without DBS. Regression analyses incorporating age, gender, and baseline weight in case or control were conducted to assess weight changes 2 years after the initial unilateral surgery. Unilateral STN DBS and staged bilateral STN DBS patients gained 3.9 ± 2.0 kg and 5.6 ± 2.1 kg versus their preoperative baseline weight (*P* < 0.001, respectively) while PD controls without DBS lost 0.8 ± 1.1 kg. Although bilateral STN DBS patients gained 1.7 kg more than unilateral STN DBS patients at 2 years, this difference was not statistically significant (*P* = 0.885). Although there was a trend toward greater weight gain in staged bilateral STN DBS patients versus unilateral patients, we found no evidence for an equivalent or synergistic increase in body weight following placement of the second DBS electrode.

## Introduction

Subthalamic nucleus deep brain stimulation (STN DBS) is an effective treatment for motor symptoms in patients with advanced Parkinson's disease (PD) who no longer respond optimally to medical therapy (Deuschl et al. 2006; Weaver et al. 2009). Although much is known about the effects of DBS on motor function, less is known about the effects of DBS on nonmotor symptoms. Stereotactic surgical treatments for advanced PD including STN and GPI (globus pallidus interna) DBS and pallidotomy have been associated with postoperative weight gain (Ondo et al. 2000; Sauleau et al. 2009). In contrast, both early and advanced PD in the absence of DBS are associated with weight loss (Chen et al. 2003; Uc et al. 2006; Bachmann and Trenkwalder, 2006). Various studies have shown that bilateral STN stimulation results in postoperative weight gain of approximately 10 kg at one year (Macia et al. 2004; Tuite et al. 2005; Novakova et al. 2007), 6 kg at an average of 16 months (Bannier et al. 2009), and 4 kg at five years postoperatively (Krack et al. 2003). Although unilateral subthalamic stimulation results in weight gain as well, comparison across studies suggest the amount of weight gain is quantitatively less (4.3 kg) at 1 year follow up compared to bilateral stimulation (Macia et al. 2004; Tuite et al. 2005; Novakova et al. 2007; Walker et al. 2009a). Little is known about changes in weight over longer time intervals or the extent of additional weight gain following staged placement of a second brain stimulator on the opposite side of the brain (Bannier et al. 2009; Walker et al. 2009a). Our clinical practice is to place a unilateral DBS electrode contralateral to the most affected hemibody, and then place another stimulator in a staged procedure on the opposite side of the brain when clinically needed. The goal of this study was to compare weight changes in PD patients who underwent both unilateral and staged bilateral STN DBS over longer time intervals (2 years) versus age-matched and disease severity matched PD controls without DBS.

## Methods

A retrospective case–control study evaluated the change in weight over a 2-year time interval following unilateral and staged bilateral STN DBS in PD. The Institutional Review Board at the University of Alabama at Birmingham approved the study. Written consent was not obtained individually from patients because the data were acquired retrospectively and deidentified. All patients were diagnosed with idiopathic PD by a movement disorder specialist using UK Brain Bank criteria (Daniel and Lees, 1993). Data on weight were reviewed from 43 consecutive patients with moderate-to-advanced PD who underwent unilateral STN DBS contralateral to their most affected hemibody. Improvements in motor function in following unilateral STN DBS in this cohort of patients at 1 year postoperatively are described in a prior study (Walker et al. 2009b). Among these patients, 25 subsequently underwent staged bilateral STN DBS when clinically necessary within 2 years of their first electrode placement. These 25 patients who had the staged procedure on the opposite side of the brain within 2 years of their initial surgery are referred to as “staged bilateral STN” patients throughout. Patients who did not undergo the staged bilateral procedure within 2 years of their initial surgery are referred to as “unilateral STN” patients, regardless of whether they have subsequently undergone the staged bilateral procedure after the 2-year follow-up period.

Weights were recorded at baseline and at 3, 6, 12, and 24 months following surgery. A second baseline weight was determined for patients who received staged placement of contralateral STN DBS, defined as the weight immediately prior to their second surgery. The staged bilateral STN DBS patients had a minimum of 3 months of subsequent follow up to evaluate weight change. Nine patients whose weight data were not available or incomplete were excluded. The initial age for the determination of baseline weight in the DBS patients was defined as the age of the subject on the day of STN DBS placement. All weights were measured during routine clinic appointments on the same electronic scale.

PD controls without DBS were identified in the University of Alabama at Birmingham Movement Disorders Registry, group matching to achieve similar age, gender, and disease severity using duration of disease and levodopa-equivalent dose (LED) per day. All controls were diagnosed and followed by a movement disorders neurologist at University of Alabama. Controls were treated with levodopa and had at least 24 months of routine clinical follow-up to establish a change in weight over time.

The LED was calculated by estimating that a 100 mg daily dose of levodopa was equivalent to the following doses of other medications: 133 mg of controlled-release levodopa, 75 mg of levodopa plus entacapone, 1 mg of pramipexole, 5 mg of ropinirole, and 10 mg of apomorphine (Deuschl et al. 2006). Controls with major medical comorbidities other than PD such as cancer or other similar chronic diseases were excluded. The baseline weight for the controls was the weight on the chart 2 years prior to the most recent clinic visit.

Descriptive statistics (means, variance, proportions) were computed on both cases and controls. All data are reported as mean ± standard error of the mean. Regression analyses incorporating age, gender, baseline weight, body mass index (BMI), and case or control were conducted to assess the final weight and weight change using SAS PROC GLM (SAS version 9. 1. 3). Chi-square statistics were used to estimate the proportion of patients who gained weight over the time period, with net weight gain and weight loss defined as any increase or decrease in body weight over the study period. Categorical analyses of change in body mass index (BMI) by National Heart Lung and Blood Institute (NHLBI) criteria (underweight ≤ 18.5, normal weight 18.5–24.9, overweight and obese ≥ 25) were conducted using chi-square statistics (National Heart, Lung, and Blood Institute, 2000). The Unified Parkinson's Disease Rating Scale (UPDRS) was measured “off” and “on” medication in DBS patients at specified intervals, but not in PD controls without DBS (Langston et al. 1992).

## Results

In this study, 43 consecutive patients with moderate-to-advanced PD underwent successful unilateral STN DBS contralateral to their most affected hemibody, and 25 (58%) of these patients underwent staged bilateral STN DBS within 2 years of their first electrode placement. The average age and duration of disease of the DBS patients were 60.6 ± 1.5 and 14.1 ± 0.90 years, respectively, and the average age and duration of disease of the PD controls without DBS were 59.7 ± 1.7 and 11.4 ± 0.97 years, respectively. Among all of the patients, 73% were male. The average latency between initial and staged electrode placement was 12.5 ± 1.6 months for patients who elected to have staged bilateral STN DBS within the 2-year interval.

**Figure 1:**
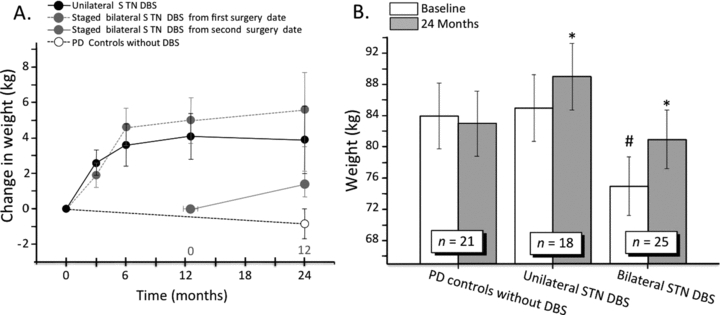
(A) Weight gain (mean ± SEM) for both unilateral and bilateral STN DBS patients is statistically significant at 24 months when compared with PD controls without DBS. A smaller weight gain occurred in the staged bilateral DBS patients following placement of the second stimulator but this difference was not statistically significant. (B) Histogram showing significant weight gain in both unilateral and staged bilateral DBS patients at 24 months (*P < 0.001 and *P* < 0.001, respectively). The patients who underwent staged bilateral DBS within 24 months of their initial electrode placement weighed significantly less than controls or unilateral patients preoperatively (#*P* < 0.0001).

Weight gain was statistically significant in both the unilateral and staged bilateral STN DBS patients at 2 years postoperatively versus controls who did not undergo DBS (*P* < 0.001, respectively). Unilateral STN DBS patients gained 3.9 ± 2.0 kg and staged bilateral STN DBS patients gained 5.6 ± 2.1 kg, while controls lost 0.8 ± 1.1 kg over the 2-year period (mean ± standard error, Fig. 1). Although the mean weight gain following staged bilateral STN DBS was 1.7 kg greater than that in the unilateral DBS patients at 2 years, this difference was not statistically significant (*P* = 0.885). The staged bilateral STN DBS patients did gain a mean of 1.4 ± 0.8 kg at 1 year after the second staged surgery versus their weight immediately prior to the second electrode placement (Fig. 1), although this change was not statistically significant (*P* = 0.119). A power calculation suggests that 87 additional DBS subjects would be required to show a significant weight gain at an average follow-up of 1 year after the staged bilateral surgery, supporting a trend for modest additional weight gain following staged placement of a second STN DBS electrode.

The majority of the weight gain in the STN DBS patients occurred in the first six months after the initial electrode placement in both the unilateral STN DBS patients and the staged bilateral STN DBS patients. There was a trend toward weight loss in the unilateral patients while weight gain persisted in the staged bilateral patients over the 1- to 2-year postoperative interval. The maximum amount of weight gained by a unilateral STN DBS case at 2 years postoperatively was 20.5 kg, and a greater than 10 kg weight gain occurred in three unilateral cases (20%). The maximum amount of weight gained by a staged bilateral STN DBS case at 2 years postoperatively was 20 kg, and greater than 10 kg weight gain occurred in four staged bilateral cases (25%). Weight gain of 10 kg or greater did not occur in any of the controls at 2 years, while weight loss occurred over a 2-year interval in 50% of the controls versus only 25% and 18% of the unilateral and staged bilateral STN DBS patients (*P* < 0.001, respectively, unilateral and staged bilateral STN DBS patients vs. controls, Table 1). Quantitative changes in weight were similar between men and women in the DBS and control groups. Male cases gained an average of 5.07 ± 1.65 kg compared to male controls who lost 0.88 ± 1.10 kg. Female cases gained an average of 4.04 ± 3.06 kg compared to a loss of -0.38 ± 2.79 kg for female controls. There were no significant differences in weight change between the cases and controls by gender (males gained 5.95 kg more between cases and controls compared to 4.42 kg for females). Additionally, no significant relationship was noted between the initial side for STN DBS surgery and weight gain.

**Table 1:** 

	Unilateral STN DBS	Bilateral STN DBS	PD controls	P value unilateral vs. controls	P value staged bilateral vs. controls
Number of patients	18	25	21	–	–
Age ± SD	60 ± 2.5	61.1 ± 1.1	59.7 ± 1.7	0.4605	0.2447
Gender proportion (M/F)	13/18 (72%)	17/25 (68%)	17/21 (81%)	0.2053	0.2229
Duration of disease (years)	10.3 ± 0.92	12.7 ±1.4	11.4 ± 0.97	0.2053	0.2229
Levodopa equivalent dose at baseline (mg/day)	1245 ± 114	1233 ± 67	1233 ± 62	0.9085	0.3712
Weight at 0 months (kg)	85.4 ± 5.5	74.5 ± 4.5	84.3 ± 3.7	0.6024	<0.0001
Proportion losing weight over 24 months	26%	18%	50%	<0.0001	<0.0001

Interestingly, we found that the patients who underwent staged bilateral surgery within 2 years of their initial electrode placement weighed an average of 10.2 kg (22.4 lbs.) less at baseline than both the patients who remained unilateral at 2 years and the PD controls without DBS (*P* < 0.0001 and *P* < 0.0001, respectively). Indeed, two patients (9%) among the staged bilateral STN DBS group were underweight by the NHLBI BMI criteria preoperatively versus none of the unilateral STN DBS patients or the controls without DBS. The staged bilateral STN DBS patients had a slightly longer duration of disease than the unilateral patients, and their preoperative motor function was slightly worse based on the UPDRS part 3 “off” (*P* = 0.0308). Only a trend toward greater preoperative disability was present in the UPDRS total score (*P* = 0.0982).

There were no remarkable correlations between preoperative tremor subscore, asymmetry index, or dyskinesia subscore and change in weight. Furthermore, there was no significant correlation between change in the UPDRS Part 3 in the “practically defined off” state (Langston et al. 1992) and the change in weight in the DBS patients. Importantly, UPDRS Part 3 “off” medication ratings were not available for the controls without DBS, therefore it was not possible to correlate changes in the UPDRS “off” and weight over time in patients with and without the DBS intervention.

## Discussion

Our clinical practice is to initially place a DBS electrode in the STN contralateral to the most severely affected side of the body and then place a second stimulator in the opposite STN in a staged fashion when clinically needed. At 2 years postoperatively, PD patients with both unilateral and staged bilateral STN DBS show sustained weight gain, and the weight of the PD controls without DBS trend downwards over the same time interval. Although the mean weight gain was greater in patients who underwent staged bilateral STN DBS over 2 years versus those who remained unilateral over 2 years, the difference in weight gain between the groups was modest and not statistically significant. We therefore found no evidence for an equivalent amount of additional weight gain or a synergistic effect of the second subthalamic stimulator on body weight. These data may be useful for patients evaluating the potential risks and benefits of both unilateral and staged bilateral STN DBS surgery for advanced PD. Our findings suggest that although weight gain likely occurs following the second surgery, it is more modest than the weight gain following the first surgery, assuming that the first surgery is performed contralateral to the more severely affected hemibody. Furthermore, the total weight gain in patients who underwent bilateral STN DBS in this study is substantially less (approximately 5 kg) than that reported in other studies in which stimulators were placed bilaterally in a single procedure (approximately 10 kg) (Macia et al. 2004; Tuite et al. 2005; Novakova et al. 2007), suggesting a potential differential effect of initial bilateral STN DBS versus staged bilateral DBS on weight gain. The magnitude of the observed changes in body weight in patients undergoing staged bilateral STN DBS surgery parallels findings from studies of motor function, suggesting that like weight gain, motor improvement following staged placement of a second DBS electrode may not be as large as that following the unilateral procedure (Samii et al. 2007).

**Figure 2:**
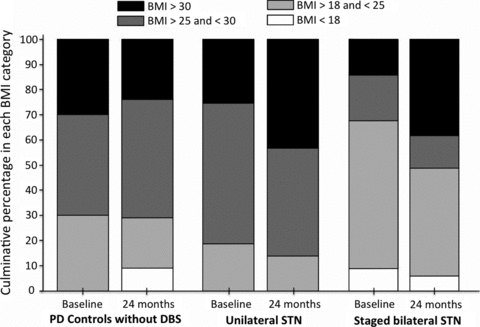
Distribution of body mass index (BMI) at the time of surgery (baseline) and at 24 months postoperatively. Patients who underwent staged STN DBS within the 24-month period were more likely to be clinically underweight by NHLBI BMI criteria.

Unexpectedly, staged bilateral STN DBS patients weighed an average of 10.9 kg less at baseline than both patients who remained unilateral for 2 years and the controls. Indeed, 9% of the staged bilateral DBS group was found to be clinically underweight by the NHLBI criteria preoperatively versus none of the unilateral DBS patients or the controls (Fig. 2). Additionally, subjects who underwent staged bilateral surgery within 2 years of their original surgery had an average duration of disease of 2 years longer than the unilateral STN DBS patients prior to surgery. These findings suggest that simple measures such as body weight and duration of disease may capture aspects of PD disability that are not as clearly reflected in clinical rating scales. Coupling our findings with other studies in patients with PD, these data are consistent with progressive weight loss over time in PD patients following diagnosis (Chen et al. 2003).

A strength of this study is that a relatively large number of DBS patients and controls without DBS were followed for 2 years after placement of the initial STN electrode. Additionally, within-subject comparisons of weight over time diminished intersubject variance. A potential limitation of this study is that the participants were not randomized to unilateral DBS, staged bilateral DBS, or medical therapy alone, and the decision to undergo staged bilateral procedure was driven by clinical necessity. Although these considerations might introduce bias into the results because of underlying differences in PD phenotype, disease severity, or other factors, we attempted to minimize such potential confounds by controlling for disease severity, duration of disease, dose of dopaminergic medications, age, and gender. Regardless, our findings are reported from DBS patients in real-world movement disorders practice, which could increase the generalizability of our results to other clinical settings.

The mechanisms underlying changes in weight in PD patients with and without DBS have been a matter of debate. In PD patients without DBS, weight loss may result from increased energy expenditure from motor symptoms, decreased caloric intake because of motor disability, and/or changes in central appetite mechanisms from medications or from PD itself (Montaurier et al. 2007). Although the mechanism for weight gain following STN DBS is also likely multifactorial, several studies argue for reduction of motor symptoms playing a significant role in postoperative weight gain. Rigidity, tremor, and bradykinesia associated with PD are relieved within seconds to minutes following activation of STN DBS at therapeutic settings (Moro et al. 2002). Calorimetric studies show similar temporal dynamics, with bilateral STN DBS decreasing the basal energy expenditure within minutes after starting therapeutic stimulation (Montaurier et al. 2007). Despite this, weight gain has not been consistently correlated with motor benefit from DBS surgery in prior studies (Macia et al. 2004; Barichella et al. 2003; Walker et al. 2009b) but see (Montaurier et al. 2007; Bannier et al. 2009). A potential confound of these studies is that they are relatively small and lack a control group without the DBS intervention. An association between change in weight and change in the UPDRS “off” medications may therefore be elusive, as the majority of the DBS patients sustain both improvement in motor function and weight gain. If a study were to evaluate changes in motor function “off” medications and weight in patients with and without DBS, it would be reasonable to expect worsening of the UPDRS “off” and relative weight loss in patients without DBS and improvements in the UPDRS “off” and weight gain in patients with DBS, increasing the likelihood of a correlation between improvement in motor function and weight gain. Another hypothesis regarding the observed weight changes is that a component of the weight gain may result from alteration of central appetite mechanisms via direct or indirect stimulation of the hypothalamic region. For instance, disruption of the melanocortin system associated with DBS therapy has been implicated in weight changes in patients with PD (Escamilla–Sevilla et al. 2011). Although our data cannot directly address this issue, weight gain has been described following both pallidotomy and globus pallidus interna (GPi) DBS, a site more anatomically remote from the satiety center (Ondo et al. 2000; Sauleau et al. 2009). There are mixed findings comparing weight changes following STN and GPi DBS, with some authors reporting greater weight gain following STN DBS and a more recent study finding no significant difference in weight change between the two targets (Sauleau et al. 2009; Locke et al. 2011). As suggested previously, other factors such as changes in dopaminergic medications may also play a role in weight changes after DBS surgery.

Regardless of whether STN DBS results in greater weight gain than what would be expected from normalizing energy expenditure from motor symptoms, the possibility has been raised that STN DBS increases cardiovascular risk and other adverse health related effects of being overweight (Bannier et al. 2009). Our data provide evidence that patients who underwent staged bilateral surgery within 2 years of their initial surgery had longer disease duration and were more likely to be clinically underweight preoperatively by NHLBI BMI criteria. Additionally, the unilateral STN DBS patients began to show a trend toward resumption of weight loss at 2 years postoperatively (Fig. 1), which agrees with pooled data from multiple studies suggesting that the initial amount of weight gain following STN DBS may not necessarily be sustained over the years following surgery (Krack et al. 2003; Macia et al. 2004; Montaurier et al. 2007; Novakova et al. 2007). Most studies associating cardiovascular risk and obesity have evaluated chronic obesity rather than more subacute weight gain. While excessive postoperative weight gain is undesirable and may present health risks in some patients, further studies should ascertain to what degree weight gain associated with DBS is sustained and how it impacts health. The results of such efforts have the potential to shift the benefit versus risk assessment for patients in whom this treatment may dramatically impact quality of life.
